# The Pathogenesis and Management of Vitiligo

**DOI:** 10.7759/cureus.75859

**Published:** 2024-12-17

**Authors:** Lama M Albelowi, Rema M Alhazmi, Sara Ibrahim

**Affiliations:** 1 Medicine, Taibah University, Al-Madinah al-Munawarah, SAU; 2 Physiology, Taibah University, Al-Madinah al-Munawarah, SAU

**Keywords:** environmental factors, immune repose, management, oxidative stress, pathogenesis, skin, vitiligo

## Abstract

Vitiligo is a common autoimmune disease that progressively destroys melanocytes in the skin, resulting in the appearance of patchy depigmentation. The aim of this review is to increase awareness towards vitiligo by providing insight on the pathogenesis and management options.

Vitiligo is an acquired pigmentary skin disease, which can appear with one or a few macules. Based on the distribution pattern, vitiligo is classified into three different types: segmental, non-segmental, and unclassified. Oxidative stress, autoimmunity, and genetic factors are the main theories contributing to the cause of vitiligo, although the exact cause remains unknown. Various management methods depend on the type, severity, and progression of the disease. This dermatological condition is prevalent globally and shows a slightly higher incidence in females. Multiple hypotheses explain the complex mechanisms of vitiligo, with current research focusing on the roles of oxidative stress, genetic predisposition, and autoimmune responses in its development. Effective treatments include topical corticosteroids and phototherapy.

## Introduction and background

Vitiligo is a pigmentary skin condition characterized by the partial absence of pigmentary cells from the epidermis, resulting in hypopigmented macules and skin patches. It can occur at any age with the highest incidence recorded in the second and third decades [1]. Globally, the prevalence of vitiligo ranges between 0.4 to 2.0% with regions of greater or lesser prevalence. In 42.3% of cases, vitiligo vulgaris was the most common type [2].

Many theories are attributed to the pathogenesis of vitiligo; however, the exact etiology is unknown and is thought to be multifactorial. Vitiligo usually presents clinically with hypopigmented macules on the body, with signs more obvious in people with dark skin.. The lesions are characterized by well-marked, oval, circular, or linear-shaped, pearly white or depigmented macules and patches, and the borders are convex, varying from a few millimeters to centimeters in size and centrifugally expanded. The variable distribution of vitiligo over the skin necessitated the disease to be classified into three different types, segmental, non-segmental, and unclassified. In most cases, the diagnosis of vitiligo is very predictable based on clinical results, although the severity of the condition requires a thorough history and examination. The intensity of the disease is determined by the affected surface area of the body. For the treatment of vitiligo, different types of topical and systemic drugs, phototherapy, and laser therapy can be used, as well as surgical therapy. Topical treatment modalities include corticosteroids, calcineurin inhibitors, and vitamin-D analogs [1].

Vitiligo is a physically disfiguring, psychologically devastating, and socially stigmatizing disease. Patients with vitiligo often face a challenging disease course, as it affects the physical appearance, and hence, it carries huge psychological and social impact. Overt and large lesions are associated with the feeling of stigma and bring about more distress [3].

Therefore, the purpose of this review article is to increase awareness about the devastating and socially stigmatizing disease by spreading knowledge about the different aspects of vitiligo including pathogenesis and the management options.

## Review

Overview

Vitiligo is a muco-cutaneous disease, caused by autoimmunity against melanocytes with a resultant hypochromic macules and patches. Lesions may be localized or scattered all over the skin. The subsequent distribution has a very detrimental effect on the quality of life [4].

Prevalence

The age of onset usually differs between males and females with a slightly greater prevalence in females and 50% of onset occurs during childhood [2]. A study at the national center for vitiligo and psoriasis in Saudi Arabia concluded that 53.5% of the cases were females and the mean age of onset of vitiligo was 17.4 years. Vitiligo vulgaris was the most common type in 42.3% of cases [5]. A retrospective review at the Hera Hospital, Makkah, concluded that 67.4% of 135 patients were females [6]. However, the worldwide prevalence of vitiligo ranges from 0.4 to 2.0%, with regions of greater or lesser prevalence [2].

Types

According to a study undertaken by the Vitiligo Global Issues Consensus Conference (VGICC) in 2011-12, vitiligo can be divided into three clinical forms: non-segmental, segmental, and unclassified or undetermined. Table 1 displays the various forms and their subdivisions [7].

**Table 1:** Different types of vitiligo with their subversions [7]

Types	Subtypes
Non-segmental vitiligo	Acrofacial
	Mucosal (more than one site affected)
	Generalized or common
	Universal
	Mixed (associated with segmental vitiligo)
	Rare forms
Segmental	Unisegmental, bisegmental or multisegmental
Unclassified or indeterminate	Focal
	Mucosal (only one site affected)

The non-segmental vitiligo (NSV) is an acquired depigmentation disorder characterized by progressive and selective destruction of melanocytes, resulting in distinctive white patches that differentiate it from other forms of leukoderma. The most widely accepted theory for the pathogenesis of NSV is an autoimmune disease. Increased oxidative stress and lower E-cadherin expression can make melanocyte damage easier [8]. 

The acrofacial subtype of NSV may affect the face, head, hands, and feet, with the perioral area and digit extremities being particularly affected. The second subtype is mucosal, which, as the title indicates, affects the oral and genital mucosa. The generalized or typical form is symmetrical macules and patches. It may impact any part of the tegument, but it is most common in the hands, fingers, and face, as well as trauma-prone regions. When 80-90% of the body surface is affected the form is called universal and it is the most common form in adulthood. The generalized or common type, on the other hand, normally comes first. The mixed form is characterized by the presence of both segmental vitiligo and NSV. Finally, rare forms include vitiligo punctata, minor and follicular. These types were also considered unclassifiable. The other main form of vitiligo is segmental vitiligo, which manifests itself as one, or rarely multiple, segments of the body demonstrating a block-like or linear patch that usually does not cross the midline. This form of vitiligo is more prevalent in children and has less association with autoimmune disease than NSV. Segmental vitiligo is a distinct vitiligo variant characterized by rapid unilateral progression within 6 to 24 months, typically associated with leukotrichia. Unlike NSV, it demonstrates limited response to systemic treatments, requiring specialized therapeutic approaches that may include targeted topical interventions, phototherapy, or localized surgical techniques to optimize depigmentation management [9].

An unclassifiable form of vitiligo is a focal form which is isolated white macules without segmental distribution. This form, however, can evolve to segmental or NSV forms.Although the above-mentioned classification is regarded as a significant step toward a formal classification of vitiligo, other studies have shown that certain types, such as acrofacial vitiligo with genital lesions, can coexist. Furthermore, the unclassifiable rare types should be under the unclassified category rather than the NSV category [7].

Pathogenesis

Oxidative Stress

Oxidative stress hypothesis suggests an imbalanced redox state of the vitiliginous skin. As a result, reactive oxygen species (ROS) such as H_2_O_2_ are generated in large quantities, with concentrations reaching one millimole [10]. At this high concentration, H_2_O_2_ leads to changes in the mitochondria and, consequently, apoptosis of the melanocytes [11]. Depigmented macules are formed when melanocytes are destroyed [12]. Melanocytes, as part of epidermal cells, are frequently subjected to environmental stressors including ultraviolet (UV) radiation and various chemicals, which can lead to an increase in ROS production. Though healthy melanocytes can tolerate these stresses, vitiligo patients' melanocytes tend to be more fragile. Melanocytes from perilesional vitiligo skin, for example, have been shown to have a dilated endoplasmic reticulum (ER) and defects in their mitochondria and melanosome composition, all of which are signs of cellular tension. In agreement with ROS hypothesis, high levels of epidermal H_2_O_2_ and a decreased level of catalase, an essential enzyme that protects cells from oxidative damage, have been observed in the skin of vitiligo patients [13].

Immune System

In vitiligo, innate immunity appears to build a bridge between adaptive immunity and oxidative stress. Early in the course of vitiligo, innate immune cells are likely to be triggered by exogenously or endogenously mediated stress signals released by melanocytes and presumably keratinocytes [14]. Genomic expression analysis of vitiligo patients' skin revealed that innate immunity is abnormally high in the local microenvironment of melanocytes, especially natural killer cells. They have been discovered infiltrating the skin of vitiligo patients, implying that natural killer cells are the first responders of melanocyte stress [15].

Exosomes were discovered to be secreted by human melanocytes in response to chemically-induced stress [14]. Those exosomes contain proteins that function as damage-associated molecular patterns and eventually deliver vitiligo target antigens to adjacent dendritic cells and cause them to mature into effective antigen-presenting cells [16-18]. The connection between adaptive and innate immunity in vitiligo seemed to be mediated by inducible heat shock protein 70 (Hsp70) which prevent apoptosis during stress [19,20]. A study done by Henning et al. reported that a new version of Hsp 70, Hsp70iQ435A, which retained the color back in a depigmented laparotomy animals, a finding that carry promising treatment for vitiligo patients [21].

As far as cellular immunity is concerned, the main culprits are the CD8+ cytotoxic T-cells. Perilesional skin biopsies revealed epidermotropic cutaneous lymphocyte antigen positive lymphocytes with a higher CD8+/CD4+ ratio, indicating that cytotoxic T-cells play a role in the pathogenesis. Interleukin-17 (IL-17) and T helper type 17 (TH17) cells have been increasingly recognized to play an important role in autoimmunity. Singh et al. recently reviewed their function in vitiligo and discovered elevated levels of IL-17 in the blood as well as the tissue samples of patients with vitiligo [22]. These findings were further substantiated by Zhou et al and Bharadwaj et al. who discovered that the levels of Thl7 cells correlated well with disease activity in generalized vitiligo [23,24]. The latter also found that the expression of IL-1ß and transforming growth factor-beta (TGF-ß) in patients with active NSV are significantly higher [24]. Several studies have found an aggregation of TH and T cytotoxic (TC) cells in the junction of the dermal and epidermal areas of vitiligo lesions, indicating the development of a silent micro-inflammatory process that destroys melanocytes, implying cell-mediated immune response activity [25].

Genetics

Although vitiligo affects just around 1% of the population, it does show signs of familial clustering [13]. According to various reports, the prevalence of vitiligo among first-degree relatives ranges from 0.14% to as high as 20% [10]. A patient's relative has a 6% chance of contracting the disease [13]. Despite this, only 23% concordance was found among monozygotic twins, implying that there is a major non-genetic component in the pathogenesis of vitiligo [10]. Therefore, the most palatable theory is that vitiligo is an autoimmune disease with a genetic basis and environmental components. Till date, no specific genetic test of vitiligo exists so far. The genetics of vitiligo is complicated since most genome-wide association studies reveal several determinants. There has been a consistent correlation with genes like DDR1, XBP1, VDR, COX2, and COMT [26].

Vitamin D Deficiency

Vitamin D nuclear receptor is found in keratinocytes, melanocytes, fibroblasts, and immune system cells of the skin, as well as in cells involved in calcium and bone metabolism. Vitamin D has a strong impact on melanocytes and keratinocytes through a variety of mechanisms. According to Vitro studies, Vitamin D3 increases melanogenesis and tyrosinase content in cultured human melanocytes and protects them from UVB-induced apoptosis, leading to vitiligo macule regimentation [10]. Various studies have shown that vitamin D analogues, such as calcipotriol and tacalcitol, may improve repigmentation in vitiligo patients, supporting the function of vitamin D in inducing repigmentation in vitiligo skin. Vitamin D has also been shown to have immunomodulatory effects by lowering the levels of different proinflammatory cytokines [27].

Environmental Factors

The environmental factors behind vitiligo are proposed by several studies. Introduction of a particular trigger from the environment in people with inherent melanocyte abnormalities may initiate the disease. This was demonstrated in a variety of manufactory employees who presented with vitiligo symptoms after being exposed to phenolic compound monobenzone [18]. Another study found a connection between vitiligo and exposure to chemicals like dyes, leather, and adhesive materials The phenols may affect melanocytes by increasing ROS production and the activation of the unfolded protein response (UPR), which triggers innate inflammation [28].

Psychological impact

Patients with vitiligo felt a persistent burden due to the unpredictability of the disease and the steps taken to conceal the disease, either through cosmetics or clothes. The major psychosocial impact is distinct in the case of patients who reported worrying about their condition during the day and were frustrated when gazing into a mirror, even with the lesions hidden. This frustration with their appearance led to a poor view of themselves, often leading to anxiety, depression, and low self-esteem. Although vitiligo does not physically pose a daunting obstacle, it has a profound detrimental effect on the well-being and quality of life of a patient unrelated to the extent of the disorder [29].

Since vitiligo is a condition visible to others, there is a psychosocial vulnerability unlike most internal illnesses, and those affected may suffer social and emotional consequences. In addition to adjusting to the physical changes they face, patients must also be mentally prepared for the likelihood of their condition spreading or worsening at any moment, creating a certain degree of uncertainty and helplessness. There is no particular distinct etiology for the condition, and this might lead patients with vitiligo to live in frequent fear and anxiety over what could cause the appearance of new lesions. The progression of the disease course, which can be gradual and episodic, is unpredictable, and to many, a source of psychological stress. Additionally, for many. In addition, studies investigating the psychological status of vitiligo patients with vitiligo reported that people suffer from feelings of disfigurement and psychological disturbance, fear, apprehension, diminished confidence, sleep disturbances, and sexual dysfunction. Various factors may contribute to and potentially worsen the disease experience, such as the extent of skin involvement, distribution, and many others [30].

Management

Treatment for vitiligo seeks to minimize mental harm while regulating autoimmune destruction to melanocytes and stimulating transmission from nearby skin and adnexal reservoirs. The three types of treatment are pharmacological, physical, and surgical, which could be combined [4].

Pharmacological Treatment

Pharmacological treatment is divided into topical and systemic. Topical treatment consists of corticosteroids, calcineurin inhibitors, and vitamin D analogues. Regarding corticosteroids in monotherapy, it is a first line treatment of localized unstable vitiligo and they can also be used in conjunction with phototherapy in generalized lesions. The best response is seen with recent and facial lesions [4].

The second type is calcineurin inhibitors, which are used to inhibit rejection in case of transplant, the first of which, cyclosporine, is not being used topically due to poor cutaneous absorption. Other calcineurin inhibitors, such as tacrolimus and pimecrolimus, showed strong absorption when used topically. A benefit of calcineurin inhibitors is that neocollagenesis is not dependent on calcineurin; therefore, there is no risk of atrophy, unlike corticosteroids, which inhibit collagen synthesis, raise the likelihood of skin atrophy, especially when used for long periods of time [7].

Finally, when melanocytes receive vitamin D, tyrosinase activity is promoted and hence melanogenesis occurs, together with the up-regulation of c-Kit as well as morphological modifications, mainly increases the number and length of the dendrites. Vitamin D causes differentiation, decreased proliferation, and the release of the proinflammatory cytokines IL-8 and IL-6 in keratinocytes, as well as an increase in IL-10 output. Both melanocytes and keratinocytes in vitiligo skin have been shown to have altered Ca++ uptake and a lower intracellular concentration of the ion, which can compromise melanogenesis by increasing the level of reduced thioredoxin, which inhibits tyrosinase [31].

Systemic corticosteroid therapy is a form of pharmacological treatment that is used in cases of disseminated vitiligo lesions that are rapidly progressing. In patients with unstable vitiligo responding to systemic corticosteroids, there was a decrease in complement-mediated cytotoxicity and levels of antibodies against melanocyte surface [7].

Melanocyte Regeneration Phototherapy

The alpha-melanocyte-stimulating hormone (a-MSH) is a natural hormone that promotes the production of melanocytes [32]. a-MSH phototherapy stimulates melanocyte regeneration and is considered as the choice of treatment for vitiligo, particularly in patients with extensive skin affection [33]. Phototherapy induces regain of pigmentation by suppressing the immune response and enhance the melanocyte stem cells to proliferate [34]. UV radiation, both in UVA and UVB spectrum, has been beneficial probably by causing immunosuppression and preventing melanocyte deformity or activating their proliferation and migration [35].

The best UV radiation used is the narrow band UVB (NBUVB). It obviates the necessity for patients to take an oral psoralen in combination with other medications, freeing them from ocular and gastrointestinal side effects. Recently, a study has investigated the role of a synthetic analogue of a-MSH, afamelanotide in subcutaneous implant together with NBUVB in vitiligo. The outcome was satisfactory except for hyperpigmentation and nausea, leading some participants to drop out of the study. The treatment resulted in quicker and high repigmentation in contrast to NBUVB monotherapy. This response was mostly seen in patients with darker tones [36].

Finally, photo-chemotherapy is a therapeutic way that involves the administration of a medication that improves the light's results. Psoralens are the most frequently used medications for treating vitiligo, The most common short-term side effects include skin and retinal phototoxicity, nausea, and headache, while long-term side effects include photoaging and a high risk of skin cancer, mostly squamous cell carcinoma which are dose-depending [37].​​​​​​​​​​

Surgical Treatment

According to a review article which was conducted by Sarkar et al., failure of pharmacological treatment indicates surgical intervention particularly in patients who suffer from a stable (non-progressive). Various surgical interventions are used in the treatment of vitiligo: split-thickness grafting, punch grafting, smash grafting, and autologous suction blister grafting. Except melanocyte culture grafting, all of these grafting procedures are simple to conduct and do not necessitate the use of complex instruments. These grafting techniques are classified as tissue grafts when an entire epidermal/dermal tissue is transplanted, or cellular grafts when individual cellular compartments are transplanted. In fact, the majority of studies comparing vitiligo grafting strategies have shown that suction blister grafting or split thickness grafting yield the best results. The benefits of partial thickness grafting over suction blistering is that it can treat a greater region of vitiligo in a single sitting. NBUVB can be used to speed up and improve the performance of partial thickness skin grafting and suction blister grafting [38].​​​​​​​​​​​​​​​​​​​​​​​​​​​​

Psychological Interventions

According to a study, half of vitiligo patients will develop lesions by the age of 20, and 25% of affected children will develop lesions by the age of 10 [39]. Despite this, there is a paucity of data on possible strategies for reducing the psychological effects in youth. Vitiligo affects not only children but also family members and caregivers, according to a number of studies [39].

Another useful intervention choice for vitiligo is camouflage. It can be learned in a single day, and the results are immediately apparent. Since vitiligo improvement may take months to years, camouflaging lesions may offer a much-needed coping strategy for patients with a highly noticeable condition. Many dermatologic disorders are used in most camouflaging trials, with comparisons of patients with vitiligo as a subset. Although this means that further study of vitiligo patients is needed, significant conclusions about its possible application can still be made. A significant number of trials have looked at the use of psychotherapy in the treatment of vitiligo. Psychotherapy offers a range of tools for detecting perceptual, mental, and behavioral signs that can then be targeted for psychotherapeutic intervention. These strategies are implemented based on the disorder of interest and the desired behavior. There are also support groups where people who have had similar experiences may gather and draw strength from one another. They also provide members a sense of belonging to a community and minimize feelings of isolation and hopelessness. Many patients experience discrimination from others and feel that their doctors, colleagues, and family do not provide sufficient support. Support groups can also be beneficial for families with young children with vitiligo who are dealing with feelings of guilt and emotional distress, financial strain, and sibling competition due to the attention focused on the child with the disease. While it is widely agreed that children's support groups are beneficial, few studies have given verifiable data. There is a lot of research on eczema patients, but to our knowledge, none has investigated the impact of support groups in children with vitiligo [40].

## Conclusions

Vitiligo represents a complex, multifactorial acquired pigmentary disorder characterized by progressive depigmentation, significantly impacting patients' psychological well-being and quality of life. Our comprehensive review synthesizes current understanding across three primary classifications: segmental, non-segmental, and unclassified variants. Key outcomes reveal multiple contributing factors including oxidative stress, autoimmune mechanisms, genetic predispositions, and environmental triggers, highlighting the intricate interplay of immunological and molecular processes. The treatment landscape demonstrates diverse therapeutic approaches ranging from topical and systemic corticosteroids to advanced phototherapy and surgical interventions, with emerging strategies targeting melanocyte regeneration and immunomodulation. Our analysis underscores the critical importance of a holistic management approach that recognizes psychological implications and emphasizes personalized treatment strategies. While significant progress has been made in understanding vitiligo, continued multidisciplinary research remains crucial for advancing comprehensive management strategies, exploring precise pathogenetic mechanisms, and developing more targeted and effective therapeutic interventions that can improve patient outcomes and quality of life.
